# A Combination of N-Terminal proB-Type Natriuretic Peptide and Myoglobin Can Predict Severe Complications After Major Non-Cardiac Surgery in Elderly Patients: A Prospective Observational Cohort Study

**DOI:** 10.3389/fmed.2021.679260

**Published:** 2021-09-27

**Authors:** Yi Zhao, Xuechao Hao, Yihao Zhu, Mingkai Chen, Mengchan Ou, Tao Zhu

**Affiliations:** ^1^Departments of Anesthesiology, West China Hospital of Sichuan University, Chengdu, China; ^2^The Research Units of West China-Chinese Academy of Medical Sciences (2018RU012), West China Hospital of Sichuan University, Chengdu, China

**Keywords:** NT-pro BNP, MYO, postoperative complications, elderly patients, risk stratification

## Abstract

**Background:** Previous studies have demonstrated that serum N-terminal proB-type natriuretic peptide (NT-proBNP) was a predictor of adverse cardiovascular outcomes after surgery. We performed a prospective study to evaluate if NT-proBNP could be a sensitive marker of overall postoperative outcomes in older patients undergoing major elective non-cardiac surgery when combined with myoglobin (MYO).

**Methods:** Two hundred and three adults aged ≥65 years were enrolled in the study. The American Society of Anesthesiologists (ASA) physical status of patients were I to IV. Blood samples would be taken before and 2 h after the surgery for each patients and NT-proBNP and MYO concentrations (NT-proBNP baseline/ 2 h and MYO baseline/ 2 h) of these samples would be measured immediately. The primary outcome was moderate to severe complications, which were based on the Clavien–Dindo Classification (CDC) scheme (≥CDC grade 3), and the secondary outcomes were major complications within 30 days after surgery. This study was registered at China Clinical Trial Registry (ChiCTR1900026223, http://www.chictr.org.cn/).

**Results:** Overall, moderate to severe complications occurred in 15 patients (7.4%) and major complications occurred in 18 patients (8.9%). Both preoperative and postoperative NT-proBNP values were independent predictors of moderate to severe complications (area under the curve (AUC), 0.820; 95% CI: 0.728, 0.912, *P* < 0.001; AUC, 0.785; 95% CI: 0.685, 0.885, *P* < 0.001). When NT-proBNP baseline and MYO-2 h were combined (NT-proBNP baseline × MYO-2 h), the predictive power was improved (AUC 0.841, 95% CI: 0.758, 0.923, *P* < 0.001).

**Conclusions:** A combination of perioperative NT-proBNP and postoperative MYO concentrations was a good predictor of postoperative complications in elderly patients who underwent major non-cardiac surgery. Using fast and dynamic tests provided by point-to-care-testing, NT-proBNP and MYO concentration measurements provided useful guidance for therapy before or soon after surgery, thus helping to reduce postoperative complications in elderly patients.

## Introduction

Postoperative death has been ranked as the third main cause of death globally (1). Although mortality and morbidity after surgery have decreased in the past decades (2), the risk for elderly patients remains unacceptably high (3, 4). The precautionary assessment of risk for elderly adults has historically focused on age and preexisting comorbidities (3, 5, 6). Examples of surgery risk-stratification tools include the American Society of Anesthesiologists (ASA) Physical Status Classification System, Acute Physiology and Chronic Health Evaluation (APACHE-II), Physiologic and Severity Score for the Enumeration of Mortality and Morbidity (POSSUM), Goldman Cardiac Risk Index (GCRI), etc. (7). However, variables in the traditional tools cannot be assessed dynamically throughout the perioperative phase (8). Therefore, a simple and fast method to enhance preoperative risk stratification is needed. The value of biomarkers in predicting perioperative morbidity and mortality has been recently investigated. An increase in the preoperative serum concentration of N-terminal proB-type natriuretic peptide (NT-proBNP) has been suggested to be a valuable predictor for major adverse cardiovascular events (MACE) after non-cardiac surgery in adult patients (9–11). In addition, both preoperative and postoperative (1 h after surgery) NT-proBNP concentrations were independent predictors of postoperative atrial fibrillation (10). Levels of NT-proBNP also increase in sepsis, cirrhosis, hyperthyroidism, and renal failure (12–14). Thus, the NT-proBNP concentration is a sensitive indicator of cardiac capacity and responded rapidly to pathological challenges. Due to the fragility of the aged cardiovascular system, it is essential to determine the predictive value of the NT-proBNP concentration for the postoperative risks of elderly patients.

Myoglobin (MYO) is a structural protein in the cytoplasm of myocardial and skeletal muscle cells that can be rapidly released into the blood once damage occurs (15, 16). MYO is commonly used for the diagnosis of myocardial injury in clinical settings (17), but it is not specific to myocardial muscle. Surgical incision will cause direct muscle injury which elevates MYO concentration. Oxidative stress or inflammatory responses can also induce damage to myocardial and skeletal muscle cells, causing a further increase in the serum MYO (18, 19). In general, MYO can be considered as an intraoperative tissue damage marker, and we hypothesized that MYO can also serve as a marker of postoperative complications. One study has demonstrated that serum analysis can predict ICU mortality and the need for renal replacement therapy (RRT) in patients after cardiac surgery (20), but there is still no unequivocal answer for major elective non-cardiac surgery.

Therefore, the present study investigated whether serum NT-proBNP and MYO concentrations could predict mortality and morbidity after major elective non-cardiac surgery in elderly patients. We also determined the clinical prognostic value when these two markers were combined. Considering the pathophysiological changes that occur during surgery, which is a composition of surgical trauma and preoperative functional response, we assumed that the combination of these two biomarkers might provide a better predictive value because NT-proBNP and MYO concentrations are associated with functional challenges and tissue damage, respectively.

## Methods

### Patients

Approval for this study (No. 199, 2020) was provided by the Ethics Committee on Biomedical Research at West China Hospital of Sichuan University, Sichuan, China (Chairperson Prof Shaolin Deng) on May 25, 2020 and registered at the China Clinical Trial Registry (ChiCTR1900026223, https://www.chictr.org.cn). This single-center, prospective observational cohort study was performed from June 2020 to November 2020. Patients eligible for inclusion were scheduled for major non-cardiac surgery (>3 h) under general anesthesia, aged ≥65 years, and had an ASA of I–IV. There was no limitation regarding gender. Patient exclusion criteria included known hematological diseases, long-term use of sedative or psychotropic drugs, an unstable mental state, and a history of allergy to anesthetics. Written informed consent was obtained from all patients or a close relative.

### Anesthesia and Surgical Procedure

All patients underwent a standard preoperative assessment, including medical history, physical examination, laboratory blood tests, and a 12-lead ECG. If further investigations were necessary, they were carried out at the discretion of the operating team. One day before surgery, responsible anesthetists were asked to make a preoperative subjective assessment of patients. Comorbidities were based on the medical history and self-reporting. Hypertension was considered to be present if the patient had previous medical documentation of hypertension and was currently taking antihypertensive medication. Coronary artery disease was defined as follows: a history of angina; myocardial infarction; positive exercise; a nuclear or echocardiographic stress test; resting wall motion abnormalities on echocardiogram; coronary angiography with evidence of ≥50% vessel stenosis; or an electrocardiogram with pathologic Q-waves in two contiguous leads. A requirement for insulin or oral hypoglycemic therapy at the time of admission for surgery was considered to be diabetes mellitus. Arrhythmias were considered to be any type of previously diagnosed arrhythmias or abnormal ECG after admission. A patient was considered a smoker if they had a history of smoking within 1 year before surgery. Evidence for chronic obstructive pulmonary disease was extracted from the primary care records and past medical history of the patient. Cerebrovascular disease was defined as a previous cerebrovascular accident or a transient ischemic attack.

All patients were routinely monitored with pulse oximetry, electrocardiography, non-invasive arterial blood pressure measurement, and the bispectral index (BIS, Covidien LLC, MA, US) in the operating room. General anesthesia was induced by intravenous propofol 1.5–2.5 mg/kg, and sufentanil 3 μg/kg, cisatracurium 0.2 mg/kg or vecuronium bromide 0.1 mg/kg was subsequently administered. After tracheal intubation, the patients were ventilated to normocapnia using an inspired oxygen fraction of 0.5 with a fresh gas flow of 2 L/min of oxygen and air. Anesthesia was maintained with an intravenous remifentanil infusion and a sevoflurane or desflurane inhalation or propofol TCI. A target BIS value of 40–60 was maintained by adjusting the dosage of the general anesthetics. At skin closure after surgery, desflurane and remifentanil were terminated. Neostigmine 20 μg/kg with atropine 10 μg/kg was administered to antagonize the residual neuromuscular block unless contraindicated. Patients were transferred to the postanesthesia care unit (PACU) or ward after tracheal extubation, which was performed after patients were confirmed to be responsive to verbal commands and to have adequate spontaneous respiratory responses. Some patients were transferred into the intensive care unit (ICU) directly. All the anesthetic or surgical procedures were administered at the discretion of the attending anesthetist or surgeon, respectively.

### NT-proBNP and MYO Test

All patients underwent venous blood sampling two times to measure NT-proBNP and MYO concentrations, just before surgery in the operating room (NT-proBNP baseline/MYO baseline) and 2 h after surgery (NT-proBNP−2 h/MYO−2 h). Blood samples were collected in EDTA evacuated tubes, immediately centrifuged, and then analyzed using a Finecare 3 plus FS-205 immunofluorescence automatic analyzer (Guangzhou Wanfu Biotechnology Co., Ltd, Guangzhou, China) in the operating room. The normal reference ranges were 0–300 pg/ml and 0–58 ng/ml for NT-proBNP and MYO, respectively.

### Patient Follow-Up

Research personnel followed patients throughout their hospital stay once a day to ascertain the presence of postoperative complications. The severity of complications was categorized using a modified CDC scheme (21). Based on CDC scores, we also used an online calculation program (www.assessurgery.com) to calculate the comprehensive complication index (CCI) for each patient.

After hospital discharge, patients were contacted by telephone 30 days after surgery to confirm the incidence of post-discharge complications. Postoperative major complications were based on a visit during their hospital stay and telephone follow-up after discharge. Mortality was defined as death within 30 days after surgery. A stay in the ICU for > 24 h was also considered to be a major complication. Acute kidney injury (AKI) was defined according to kidney disease improving global outcomes (KDIGO) criteria (2012) (22). Cardiovascular events included the development or aggravation of congestive heart failure, acute myocardial infarction, or arrhythmias. Postoperative delirium (POD) was diagnosed based on the confusion assessment method-ICU (23). The primary outcome in the present study was moderate to severe complications (≥CDC grade 3) and the secondary outcomes were postoperative major complications within 30 days after surgery including death, an ICU stay > 24 h, MACE, respiratory failure, stroke, AKI, and POD > 24 h.

### Statistical Analysis

The sample size calculation was based on comparing the area under the curve (AUC) of the receiver-operating characteristic (ROC) curves using MedCalc (Version 19). Assuming an outcome event rate of 8% (based on our previous study), a moderately good AUC of 0.75 for blood NT-proBNP or MYO concentrations, a sample size of 188 patients had 90% power to detect a clinically relevant difference in AUC values (two-sided alpha of 0.05). To account for 10% of patients who may have been lost to follow-up, we aimed to recruit a total of 206 patients to the study. The missing data were excluded.

Statistical analysis was performed using SPSS Version 15 software (SPSS, Chicago, IL, US). All statistical tests were two-sided, and significance was assumed at a value of *P* < 0.05. Comparison of interval data was performed using the independent sample *t*-tests, the Mann–Whitney, or the Kruskal–Wallis tests when appropriate. NT-proBNP/MYO concentrations and NT-proBNP × MYO concentrations were entered as both continuous and categorical variables at different levels for analyses. The ROC curve analysis was performed to identify the predictive values of NT-proBNP and MYO concentrations for postoperative complications. The performance of continuous variables was analyzed with ROC curves, with the AUC being calculated. Cutoff values were evaluated based on the Youden index. Relative risk (RR) and 95% confidence intervals (CIs) were determined using a chi-squared test.

## Results

Between June 2020 and November 2020, 603 patients met the study inclusion criteria. Only 224 patients provided consent to participate in the study, and 12 patients had their surgery unexpectedly canceled. Finally, 212 patients in our hospital were enrolled in the study and 203 (96.5%) completed their 30-day follow-ups.

According to our protocol, blood samples were taken both in the operating room before surgery and in the PACU 2 h after surgery for each patient. For those patients transferred directly into the ICU or wards, without entering PACU, blood samples were taken there.

The median age of included patients was 72 years (67, 76) of which 26.6% were female. The demographic characteristics and preoperative comorbidities of the patients are shown in Table 1. Most patients underwent major abdominal, urological, thoracic, or orthopedic procedures. The incidence of complications within 30 days of surgery is shown in Table 2. The postoperative complications of 15 (7.4%) patients were ≥grade 3 based on the CDC scheme. The incidence of major complications was 8.9% (18 patients).

**Table 1:** Participant baseline characteristics and type of surgery (*n* = 203).

**Characteristics**	**Value, mean ± SD/median (quartile)/n (%)**
Age (years)	72 (67, 76)
Female sex	54 (26.6%)
Height (cm)	161.1 ± 7.2
Weight (kg)	60.9 ± 7.2
**Comorbidities**	
Hypertension	90 (44.3%)
Coronary artery disease	8 (3.9%)
Arrhythmias	15 (7.4%)
Diabetes mellitus	28 (13.8%)
Current or recent smoker	43 (21.2%)
COPD	15 (7.4%)
Cerebrovascular disease	6 (3.0%)
**ASA—PS**	
Class 2	104 (51.2%)
Class 3	99 (48.8%)
**Motion equivalent**	
>6 MET	41 (20.2%)
3–6 MET	146 (71.9%)
<3 MET	10 (5.0%)
**Surgery**	
Open surgery	124 (61%)
Procedure type Hepatobiliary Gastrointestinal Pancreatic Urological	40(19.7%) 93(45.8%) 16(7.9%) 54(26.6%)

*COPD, chronic obstructive pulmonary disease; ASA—PS, American Society of Anesthesiologists Physical Status; MET, metabolic equivalent*.

**Table 2:** Characteristics of 30-day complications after surgery and perioperative level of NT-proBNP, MYO (*n* = 196).

**Characteristics**	**Value, *n* (%)**
Complications ≥CDC grade 2	118 (58.1%)
Complications ≥CDC grade 3	15 (7.4%)
Major complications	18 (8.9%)
Mortality	1 (0.5%)
ICU stay > 24 h	6 (3.0%)
Acute kidney injury	8 (3.9%)
Cardiovascular events	5 (2.5%)
Respiratory failure	6 (3.0%)
Cognitive impairment > 24 h	7 (3.4%)
CCI score	22.0 (12.2, 25.7)
NT-proBNP baseline (pg/mL)	279.7 (145.2, 311)
NT-proBNP−2 h (pg/mL)	166.3 (151.2, 326.9)
MYO Baseline (ng/mL)	39.1 (19.9, 37.3)
MYO-2 h (ng/mL)	85.5 (48.4, 171.0)

*CDC, Clavien-Dindo Classification; CCI, Comprehensive Complication Index; NT-proBNP, N-terminal pro-B-type natriuretic peptide; MYO, myoglobin; NT-proBNP baseline/MYO baseline, plasma concentration of NT-proBNP/MYO before surgery; NT-proBNP−2 h/MYO-2 h, plasma concentration of NT-proBNP/MYO 2 h after surgery*.

**Figure 1:**
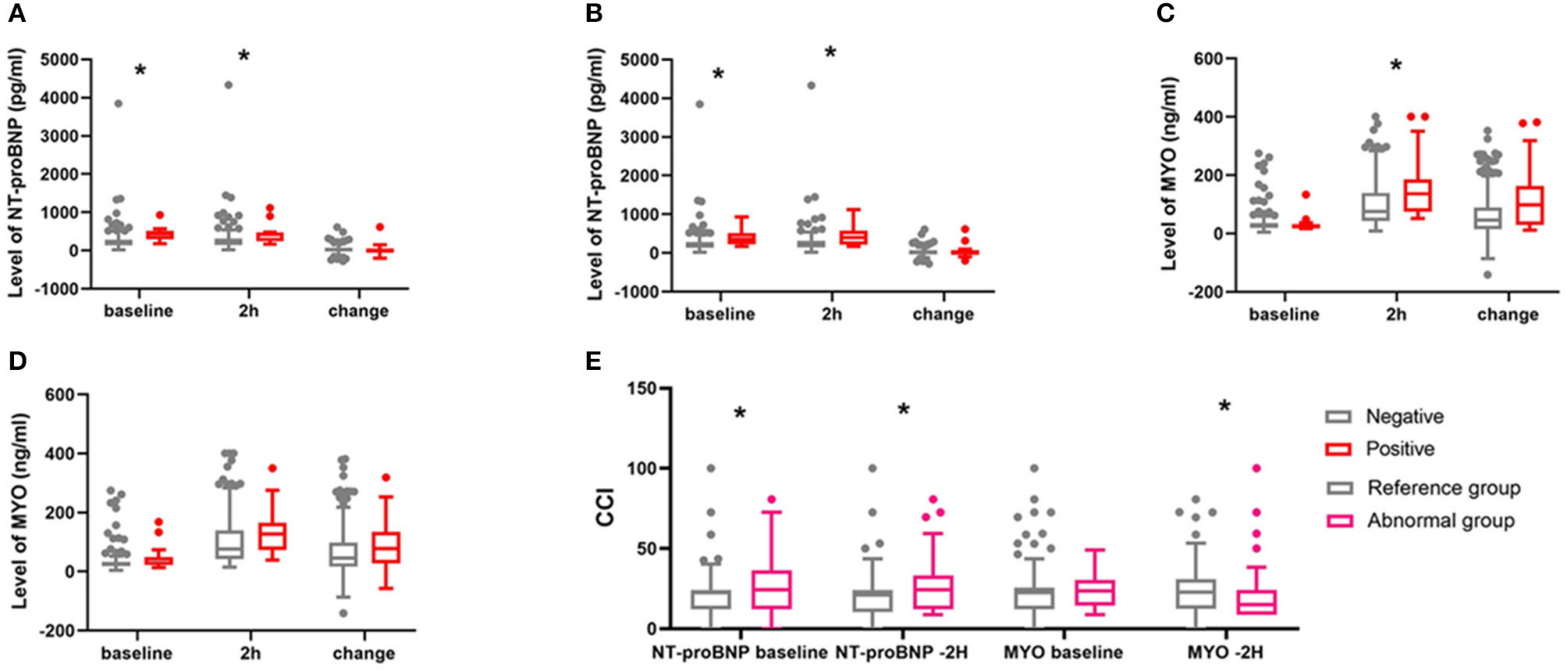
Comparison of peri-operative concentrations of NT-proBNP and MYO between complication positive and complication negative patients and CCI between patients with reference and abnormal concentrations. **(A)** NT-proBNP for complications ≥CDC grade 3, *indicates *P* < 0.001. **(B)** NT-proBNP for major complications, *indicates *P* < 0.001. **(C)** MYO for complications ≥CDC grade 3. *P = 0.015. **(D)** MYO for major complication. **(E)** comparison of CCI between patients with reference concentrations and abnormal concentrations of NT-proBNP and MYO. *P < 0.005.

As shown in Figure 1, for patients who developed complications ≥CDC grade 3 or major complications, the concentrations of NT-proBNP at baseline were higher than for patients without corresponding complications (*P* < 0.01). No difference was found in the perioperative NT-proBNP mean concentration among complication-positive or negative patients (*P* > 0.05). The levels of MYO at 2 h in patients who developed ≥CDC grade 3 were higher than in patients without corresponding complications (*P* < 0.01). But for patients who developed major complications, there was no difference in the MYO baseline, MYO at 2 h, or changes in the MYO concentration. The CCI score was lower in patients with reference concentrations of NT-proBNP or MYO than in those with abnormal concentrations (*P* < 0.005).

**Figure 2:**
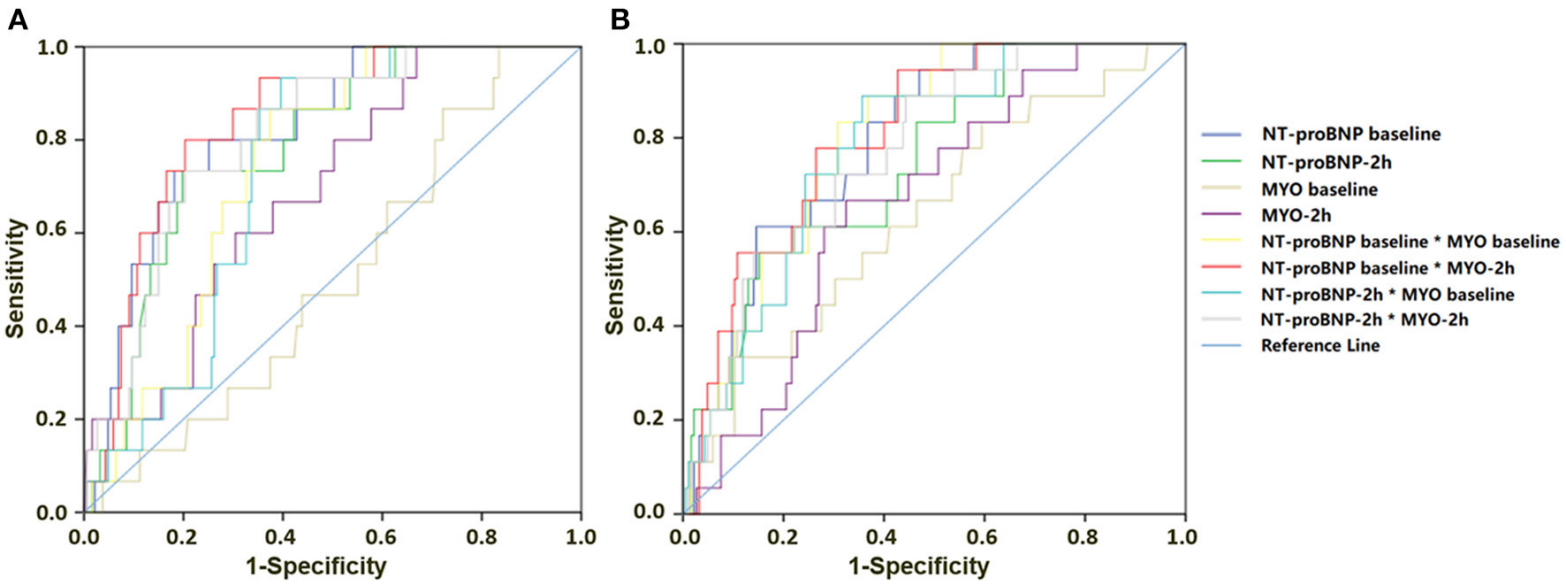
Results of ROC analysis for NT-proBNP and MYO predicting complications within 30 days after surgery**. (A)** NT-proBNP and MYO concentrations for predicting complications ≥CDC grade 3. **(B)** NT-proBNP and MYO for predicting major complications.

The ROC analysis showed the predictive value of NT-proBNP baseline concentrations, NT-proBNP and MYO at 2 h for complications ≥grade 3, or major complications (Figure 2; Table 3). For complications ≥CDC grade 3, the AUC of NT-proBNP baseline or the NT-proBNP at 2 h were 0.820 and 0.785, respectively (*P* ≤ 0.001). The cutoff concentration of the NT-proBNP baseline was 312 pg/ml and it exhibited the best combined sensitivity (0.733) and specificity (0.818). The cutoff concentration of NT-proBNP at 2 h was 340 pg/ml, the sensitivity and specificity of which were 0.733 and 0.802, respectively. The AUC of combination NT-proBNP and MYO using NT-proBNP baseline × MYO at 2 h was 0.841 (95% CI: 0.758, 0.923, *P* < 0.001). The cutoff of NT-proBNP baseline × MYO at 2 h was 32,828 and had the best combined sensitivity (0.800) and specificity (0.797). For major complications, the AUC of the BNP baseline and the NT-proBNP concentration at 2 h were 0.794 (*P* ≤ 0.001) and 0.745 (*P* = 0.001), respectively. The results of ROC analysis are shown in Figure 2; Table 3.

**Table 3:** Predictive performance of perioperative level of NT-proBNP and MYO for 30-day complications.

**Variables**	**AUC (95% CI)**	**Cutoff value**	**Sensitivity**	**Specificity**	***P* value**
**Complications ≥CDC grade 3**					
NT-proBNP baseline	0.820 (0.728–0.912)	312	0.733	0.818	<0.001
NT-proBNP−2 h	0.785 (0.685–0.885)	340	0.733	0.802	<0.001
MYO Baseline	0.505 (0.373–0.638)				0.945
MYO-2 h	0.690 (0.571–0.808)				0.015
NT-proBNP baseline × MYO baseline	0.743 (0.653–0.833)	5831	0.867	0.374	0.002
NT-proBNP Baseline × MYO-2 h	0.841 (0.758–0.923)	32828	0.800	0.797	<0.001
NT-proBNP−2 h × MYO baseline	0.728 (0.640–0.817)	6299	0.933	0.604	0.003
NT-proBNP × MYO-2 h	0.809 (0.715–0.903)	37767	0.733	0.797	<0.001
**Major Complications**					
NT-proBNP baseline	0.794 (0.707–0.881)	199	0.944	0.430	<0.001
NT-proBNP−2 h	0.745 (0.637–0.853)	393	0.556	0.849	0.001
MYO baseline	0.634 (0.502–0.766)				0.640
MYO-2 h	0.665 (0.555–0.775)				0.021
NT-proBNP baseline × MYO baseline	0.799 (0.719–0.880)	7283	0.833	0.692	<0.001
NT-proBNP baseline × MYO-2 h	0.808 (0.722–0.893)	16652	0.944	0.573	<0.001
NT-proBNP−2 h × MYO baseline	0.778 (0.686–0.870)	7037	0.889	0.643	<0.001
NT-proBNP−2 h × MYO-2 h	0.771 (0.675–0.868)	17945	0.889	0.557	<0.001

*CDC, Clavien-Dindo Classification; AUC, area under the curve; NT-proBNP, N-terminal pro-B-type natriuretic peptide; MYO, myoglobin; NT-proBNP baseline/MYO baseline, plasma concentration of NT-proBNP/MYO before surgery; NT-proBNP−2 h/MYO-2 h, plasma concentration of NT-proBNP/MYO 2 h after surgery*.

Relative risk values were also calculated to identify the risk of different concentrations of the NT-proBNP baseline and the NT-proBNP baseline × MYO-2 h (Table 4). For complications ≥CDC grade 3, the RR of NT-proBNP baseline in the range 300–600 pg/ml was 8.13 (95% CI: 2.950, 22.727; *P* < 0.001) and the RR of the NT-proBNP baseline ≥600 pg/ml was 1.379 (95% CI: 0.200, 9.434; *P* = 0.746); the RR of the NT-proBNP baseline × MYO-2 h between 30,000 and 60,000 was 2.488 (95% CI: 0.907, 6.803; *P* = 0.074) and the RR of the NT-proBNP baseline × MYO-2 h ≥60,000 was 7.092 (95% CI: 2.710, 18.519; *P* < 0.001). For major complications, the RR of the NT-proBNP baseline between 300 and 600 pg/ml was 3.257 (95% CI: 1.376, 7.752, *P* = 0.006) and the RR of the NT-proBNP baseline ≥600 pg/ml was 3.861 (95% CI: 1.331, 11.236; *P* = 0.016); the RR of the NT-proBNP baseline × MYO−2 h ≥60,000 was 6.230 (95% CI: 2.473, 15.695; *P* < 0.001).

**Table 4:** Association between NT-proBNP baseline/(NT-proBNP baseline × MYO-2 h) and 30-day complications.

	**NT-proBNP baseline threshold, (pg/mL) (*****n*** **= 196)**				**(NT-BNP baseline × MYO-2 h) threshold, (*****n*** **= 136)**			
	**<150**	**150 to <300**	**300 to <600**	**≥600**	**<16,000**	**16,000 to <30.000**	**30,000 to <60,000**	**≥60,000**
Patients, *n* (%)	63 (31.0%)	90 (44.3%)	40 (19.7%)	10 (4.9%)	103(50.7%)	41 (20.2%)	34 (16.7%)	25 (12.3%)
Complications ≥CDC grade 3	0/63	4/90	10/40	1/10	1/103	2/41	5/34	7/25
Risk ratios	–	0.456 (0.150, 1.385)	8.13 (2.950, 22.727)	1.379 (0.200, 9.434)	0.069 (0.009, 0.518)	0.608 (0.143, 2.509)	2.488 (0.907, 6.803)	7.092 (2.710, 18.519)
*P* value	–	0.152	<0.001	0.746	<0.001	0.491	0.074	<0.001
Major complications	0/63	7/90	8/40	3/10	1/103	5/41	5/34	7/25
Risk ratios	–	0.799 (0.323, 1.976)	3.257 (1.376, 7.752)	3.861 (1.331, 11.236)	0.057 (0.008, 0.421)	1.520 (0.573, 4.020)	1.912 (0.730, 5.010)	6.230 (2.473, 15.695)
*P* value	–	0.626	0.006	0.016	<0.001	0.401	0.189	<0.001

*CDC, Clavien-Dindo Classification; NT-proBNP, N-terminal pro-B-type natriuretic peptide; MYO, myoglobin; NT-proBNP baseline, plasma concentration of NT-proBNP before surgery; MYO-2 h, plasma concentration of MYO 2 h after surgery*.

## Discussion

The present study demonstrated that preoperative the NT-proBNP concentration was a valuable predictor of postoperative moderate-to-severe complications and also major complications. When combined with postoperative MYO concentration measurements, the predictability was further improved. Our study extends the findings of other cohort studies in this research area, most of which focused on the relationship between preoperative NT-proBNP concentrations with associated postoperative MACE or mortality (3, 9, 24). Here, we concentrated on composite complications after surgery, and our findings revealed the prognostic value of NT-proBNP and MYO concentrations.

In the present study, the AUC of the NT-proBNP baseline and NT-proBNP baseline × MYO-2 h were 0.820 and 0.841, respectively, in predicting moderate to severe complications. The previous study of Payne et al. found the AUC was 0.792 in predicting all-cause mortality 1 year after major non-cardiac surgery in 345 patients (25). In another study, the AUC of NT-proBNP in predicting in-hospital moderate or severe complications was 0.72 (26). Breidthardt et al. demonstrated that the AUC for the prediction of long-term cardiac events by BNP was 0.71 in 270 patients (27). Both the AUC of NT-proBNP baseline and NT-proBNP baseline × MYO-2 h in our study were higher than reported in previous studies, perhaps because we focused on older patients who underwent major non-cardiac surgery and who were likely more vulnerable to surgical trauma. Then, related studies about MYO concentrations revealed that it could be a sensitive indicator of myocardial damage after surgery (28) and a valid indicator of the extent of muscle damage (29). The combination of the two markers took both preoperative and intraoperative statuses into consideration, and the outcomes indicated this combination had a superior prediction value in elderly patients.

According to a previous study, an elevated concentration of plasma NT-proBNP before or soon after thoracic surgery (1 h) for lung cancer was a strong independent predictor for the occurrence of atrial fibrillation (10). However, in our study, the NT-proBNP−2 h had a lower predictive power when compared with the NT-proBNP baseline. Since it was different from the MYO-2 h concentration, which was always elevated after surgery, the NT-proBNP−2 h concentration was lower than baseline in almost half of the patients. On the one hand, intraoperative infusion may partly account for this outcome. Blood loss, blood infusion, and fluid infusion during different types of surgery would most likely decrease the NT-proBNP concentration in plasma. On the other hand, NT-proBNP concentrations could not respond rapidly enough to surgical challenges within a few hours, while MYO can be released directly into the blood once tissue damage has occurred.

Another interesting finding in our study was that the AUCs of the MYO baseline and MYO-2 h were 0.505 and 0.690, respectively for complications ≥CDC grade 3. Contrary to our expectations, MYO alone had no value in predicting postoperative complications. In fact, previous studies have proven that MYO level was a potential biomarker for evaluating sepsis severity and poor outcomes, and the area under the ROC curve was 0.824 (30). Also, the MYO concentration reflected the severity of illness and could predict the survival rate in critically ill patients (31). In patients after cardiac surgery, serum MYO was associated with ICU mortality and the need for RRT (the areas under the ROC curve were 0.81 and 0.87, respectively) (26). We believe a lack of specificity may account for this result. Numerous factors during surgery, including organ damage, sepsis, release of tumor necrosis factor in cancer surgery, metabolic status, and the stress response, accelerate the release of MYO, which confuses the biochemical picture. However, MYO can improve the prognostic value of NT-proBNP when combined with it. We believe that the underlining reason was the synchronous rise of these two markers as a strong indication of global tissue and functional damage.

One limitation of the present study was the RR value of NT-proBNP baseline ≥600 for CDC ≥ 3 was 1.379 (*P* = 0.746), which suggested that patients with NT-proBNP baselines ≥600 had a lower risk than other patients. But we thought the predictive power was underestimated because the sample size was relatively small. Also, the joint predictive power of the NT-proBNP baseline and MYO-2 h may have been underestimated. In addition, as the sample size was relatively small, the number of patients with events (moderate to severe complications or major complications) was also small. Large sample studies are needed to confirm the outcomes of the present study.

In conclusion, our study revealed the predictive value of NT-proBNP and MYO concentrations for risk assessment of postoperative mortality and morbidity in elderly patients who underwent non-cardiac surgery. Using the fast and dynamic tests provided by POCT facilitates NT-proBNP and MYO concentration measurements, which should be considered for the routine preoperative assessment of patients exhibiting moderate-to-severe postoperative complications. Future studies are warranted to evaluate whether NT-proBNP and MYO concentrations can guide intraoperative and early postoperative management of elderly patients, thus improving their clinical outcomes after surgery.

## Data Availability Statement

The raw data supporting the conclusions of this article will be made available by the authors, without undue reservation.

## Ethics Statement

Ethical approval for this study (No. 199, 2020) was provided by the Ethics Committee on Biomedical Research, West China Hospital of Sichuan University, Sichuan, China (Chairperson Prof Shaolin Deng) on 25 May 2020 and registered at China Clinical Trial Registry. The patients/participants provided their written informed consent to participate in this study.

## Author Contributions

YZ: conceptualization, methodology, and writing the original draft. XH: conceptualization, methodology, and software. YZ and MC: visualization and investigation. MO: data curation. TZ: supervision. All authors contributed to the refinement of the study protocol and approved the final manuscript.

## Funding

This study was supported by the National Key R&D Program of China (Beijing, China) (Grant No. 2018YFC2001800); the CAMS Innovation Fund for Medical Sciences (grant number 2019-I2M-5-011); the Science and Technology Department of Sichuan Province, China (Grant No. 2020YJ0052); the China Postdoctoral Science Foundation (grant number 2019M663507); the Post-Doctor Research Project, West China Hospital, Sichuan University (project number 19HXBH064); the National Clinical Research Center for Geriatrics, West China Hospital of Sichuan University (Grant No. Z2018A02); and the 135 Project for Disciplines of Excellence, West China Hospital, Sichuan University (Grant No. ZYJC18010).

## Conflict of Interest

The authors declare that the research was conducted in the absence of any commercial or financial relationships that could be construed as a potential conflict of interest.

## Publisher's Note

All claims expressed in this article are solely those of the authors and do not necessarily represent those of their affiliated organizations, or those of the publisher, the editors and the reviewers. Any product that may be evaluated in this article, or claim that may be made by its manufacturer, is not guaranteed or endorsed by the publisher.
